# Policy implications for awareness gaps in antimicrobial resistance (AMR) and antimicrobial use among commercial Nepalese poultry producers

**DOI:** 10.1186/s41256-021-00187-2

**Published:** 2021-02-06

**Authors:** Anastasia S. Lambrou, Gabriel K. Innes, Laura O’Sullivan, Himal Luitel, Rebanta K. Bhattarai, Hom B. Basnet, Christopher D. Heaney

**Affiliations:** 1 Department of International Health, Johns Hopkins Bloomberg School of Public Health, Baltimore, MD USA; 2 Department of Epidemiology, Rutgers School of Public Health, Piscataway, NJ USA; 3 Department of Environmental Health & Engineering, Johns Hopkins Bloomberg School of Public Health, Baltimore, MD USA; 4 Center for Biotechnology, Agriculture and Forestry University, Rampur, Chitwan Nepal; 5 Department of Veterinary Microbiology and Parasitology, Agriculture and Forestry University, Rampur, Chitwan Nepal; 6 Department of Epidemiology, Johns Hopkins Bloomberg School of Public Health, Baltimore, MD USA

**Keywords:** Antimicrobial resistance, Withdrawal period, Poultry, Nepal, One health, Health policy

## Abstract

**Background:**

Nepal’s poultry industry has increased with a growing middle class, which has translated to an increase in antimicrobial consumption and thus a rise in antimicrobial resistance (AMR). Describing and understanding antimicrobial use practices among commercial poultry producers in Nepal may help minimize the risks of AMR development in both humans and animals and determine the effectiveness of relevant policies.

**Methods:**

From July to August 2018, poultry farmers were randomly recruited from Nepal’s Chitwan District to participate in a cross-sectional study. The lead producer in each poultry operation was administered a quantitative structured-survey via a 30-min interview. Participants were asked to provide demographics, production practices, and knowledge about their antimicrobial use practices. Descriptive data analysis was performed to obtain frequencies and compare practices.

**Results:**

In total, 150 commercial poultry producers of whom raised between 300 and 40,000 birds completed the interviews. Only 33% (*n* = 49) of producers reported knowing what AMR was, and among them only 50% (*n* = 25) consulted a veterinarian for treatment options. Antimicrobial administration for growth promotion was still employed by 13% of poultry producers. Similarly, critically important antimicrobial drugs, specifically colistin, were identified at 35% of participating operations. Producers reported low overall understanding and compliance of withdrawal periods (*n* = 41; 27%), which may result in both AMR development and adverse health reactions among consumers who ingest antimicrobial residues. Although Nepal has publicized antimicrobial use policies and awareness campaigns to instill healthy production practices, most producers (82%) were unaware of them.

**Conclusion:**

Many Nepalese poultry producers lack overall antimicrobial use and AMR awareness, which is evidenced by low antimicrobial withdrawal period compliance, use of antimicrobials for growth promotion, and the sustained use of critically important antimicrobials. Improved outreach and educational capacities, paired with increased veterinary resources and extensive monitoring in operations and retail meat products, may increase AMR awareness and policy enforcement.

## Introduction

Described as one of the most significant public health crises of our time, antimicrobial resistant organisms contribute to over 700,000 global deaths annually, a number estimated to reach 10 million by 2050 [1, 2]. Antimicrobial resistance (AMR) has been more pronounced in low- and middle-income countries (LMIC) and is projected to rise in the next decades [3]. The growing LMIC health burdens from AMR infections may be due to increased antimicrobial consumption in both human and animal sectors [4, 5]. It is estimated that animal agriculture purchases up to 73–100% more antimicrobials than human medicine, which is aligned with a rising middle class that demands animal-based protein [6–8]. The Federal Democratic Republic of Nepal (henceforth Nepal) fits this dual growth and has intensified the animal agriculture sector—notably in the Chitwan district. This district produces 40% of the nation’s egg supply and is dovetailed with increases in antimicrobial drug procurement, which has increased by 50% between 2008 and 2012 [9, 10]. The veterinary capacity has not caught up with the animal agriculture expansion. Veterinarians have been estimated to only prescribe roughly 30% of antimicrobials in Nepal, indicating a lack of veterinary oversight on the remaining antimicrobials used, consequent inappropriate usage, and subsequent emergence of antimicrobial resistance [11].

Nepal has responded to injudicious antimicrobial use in the animal agriculture sector through policy adoption that aims to decrease indiscriminate antimicrobial use. For example, the Ministry of Livestock Development’s (MoLD) banned the importation of feed enriched with antimicrobials to indirectly reduce growth promotion practices. Nepal has also established a National Action Plan for Antimicrobial Resistance (NAP-AMR), a multi-sectoral committee focused on antimicrobial stewardship for both human and animal sectors [12]. However, veterinarians have little oversight over antimicrobial drugs and little surveillance strategies and data exist to capture the antimicrobial use practices and AMR development trends. It is critical to understand antimicrobial use practices of commercial poultry producers in Nepal to minimize the risks of AMR development in both humans and animals and to evaluate whether current policies and initiatives promote judicious antimicrobial use. This research aimed to evaluate antimicrobial knowledge and antimicrobial use among the Chitwan District’s local poultry producers that will provide key elements to evaluate Nepal’s current antimicrobial use policies and inform future risk communication and stakeholder training programs.

## Methods

### Study design

A cross-sectional survey of commercial poultry producers was performed in Chitwan District, Nepal between July and August 2018. This study served as one facet of the EcoHealth Net funded One Health Collaboration between the Veterinary School of the Agriculture and Forestry University in Rampur, Chitwan Province, Nepal and the Johns Hopkins Bloomberg School of Public Health in Baltimore, Maryland, United States. The study staff included 12 veterinary undergraduate and postgraduate students from the Agriculture and Forestry University. All study staff were trained in interview administration techniques to ensure the survey questions were asked in a standardized manner and to reduce interviewer and recall biases. Pilot interviews were also conducted at poultry farms not enrolled in the study to further train study staff and monitor and evaluate survey administration.

### Commercial poultry producer enrollment

In 2018, the Nepalese Department of Health Services surveyed Chitwan District poultry operations through a national census structure. The study staff leveraged census information to enroll registered poultry operations through simple random sampling. Study staff interviewed the leadership staff of each operation on-site. The study’s sample size was calculated using a two-sided single proportion equation with the assumption that 50% of farmers in the study have awareness of AMR using the Epitools Epidemiologic Calculator [13]. A sample size of 97 farmers was needed to identify the proportion of 50% with 95% confidence and 10% absolute precision. The sample size goal was exceeded by enrolling 150 farmers and their farms.

### Inclusion criteria

Only consenting operational commercial poultry farms in Chitwan District from July to August 2018 were included in the study population. Within this study, commercial poultry operations were defined as enclosed broiler or layer farms that produced at least 100 chickens, ducks, or turkeys for the commercial market. Poultry operations without a functioning telephone number could not be contacted and were thus excluded. If a consenting facility was no longer operational, a direction was chosen at random on-site to identify and recruit an unenrolled entity. All enrolled operations consented to participation and owners were informed that their participation would not impact their commercial farm registration status. The Institutional Review Board of the Agriculture and Forestry University in Rampur, Chitwan Province, Nepal, approved this study.

### Survey tool

A quantitative structured-survey was administered to each operation’s lead producer through a 30-min interview. The survey tool was developed to answer the primary and secondary research questions using dichotomous and categorial responses on antimicrobial use and AMR knowledge and practices. Participants were also asked open-ended questions to report all antimicrobials they administer during the poultry production process and were requested to present the antimicrobial vessels for clarification and validation. All reported and presented antimicrobial drugs were categorized by class. The survey tool was further refined after a one-week piloting process to increase question clarity and cultural appropriateness.

### Statistical analysis and mapping

AMR knowledge and husbandry practices were analyzed via descriptive statistics. The statistical data analysis was conducted in STATA 14.0 (StataCorp. 2019. Stata Statistical Software: Release 14. College Station, TX: StataCorp LLC). The global positioning system (GPS) point for each operation was recorded prior to the interview. Each GPS point was collected using the Google Maps mobile application. Operations that could not be visited in person were geocoded using Google Maps (Google. Google maps. 2019 ed.: MapData sciences. 2019) and registered physical addresses. The mapping of farms enrolled in the study was produced using ArcGIS (ArcGIS [GIS Software]. Version 10.0 Redlands, CA: Environmental Systems Research Institute, Inc., 2010).

## Results

### Commercial poultry producer and farm demographics

A total of 150 poultry producers from 149 commercial chicken operations and one commercial turkey operation (Tables S1–2) were recruited into the study. Most of the respondents included in the study were male (68%), of Brahmin Caste (52%), and completed at least secondary education (65%). The commercial poultry farms varied in farm size between 300 and 40,000 birds with a mean of approximately 3000 birds. The majority of producers raised layers (66%) as compared to broilers (33%) or both layers and broilers (1%).

### Antimicrobial resistance (AMR) knowledge

Most (67%) surveyed commercial poultry producers had little or no knowledge of AMR (Table 1). Farmers with previous AMR knowledge (33%) reported that they had received AMR education from multiple sources, including veterinarians (51%) and “occupational experience” (31%). “Occupational experience” included firsthand experience and secondhand accounts from local producers’ experiences with AMR from refractory disease. The majority (92%) of individuals with previous AMR knowledge acknowledged that AMR resulting from antimicrobial use in poultry is a threat to human health.

**Table 1:** Commercial poultry farmer antimicrobial resistance (AMR) knowledge in Chitwan District, Nepal, *N* = 150 farmers

Antimicrobial Resistance Knowledge	Farmer No. (%)
**Knowledge of AMR previous to study**	
Yes	49 (32.7)
No	101 (67.3)
Don’t know	0 (0.0)
**Sources of AMR knowledge**	
Veterinarians	25 (51.0)
Occupational experience	15 (30.6)
Community members	8 (16.3)
Newspaper	8 (16.3)
Social media	5 (10.2)
Television	5 (10.2)
Physicians/hospital	4 (8.2)
Radio	2 (4.1)
**Is AMR a serious threat?**^**ab**^	
Yes	44 (91.7)
No	4 (8.3)
**Can antibiotic use in poultry impact human health?**^**a**^	
Yes	49 (100.0)
No	0 (0.0)
**Knowledge of the Nepalese government’s antibiotic use policies**	
Yes	27 (18.0)
No	123 (82.0)

^a^Farmers that responded that they had knowledge of AMR previously

^b^Missing data from one farmer

**Fig. 1:**
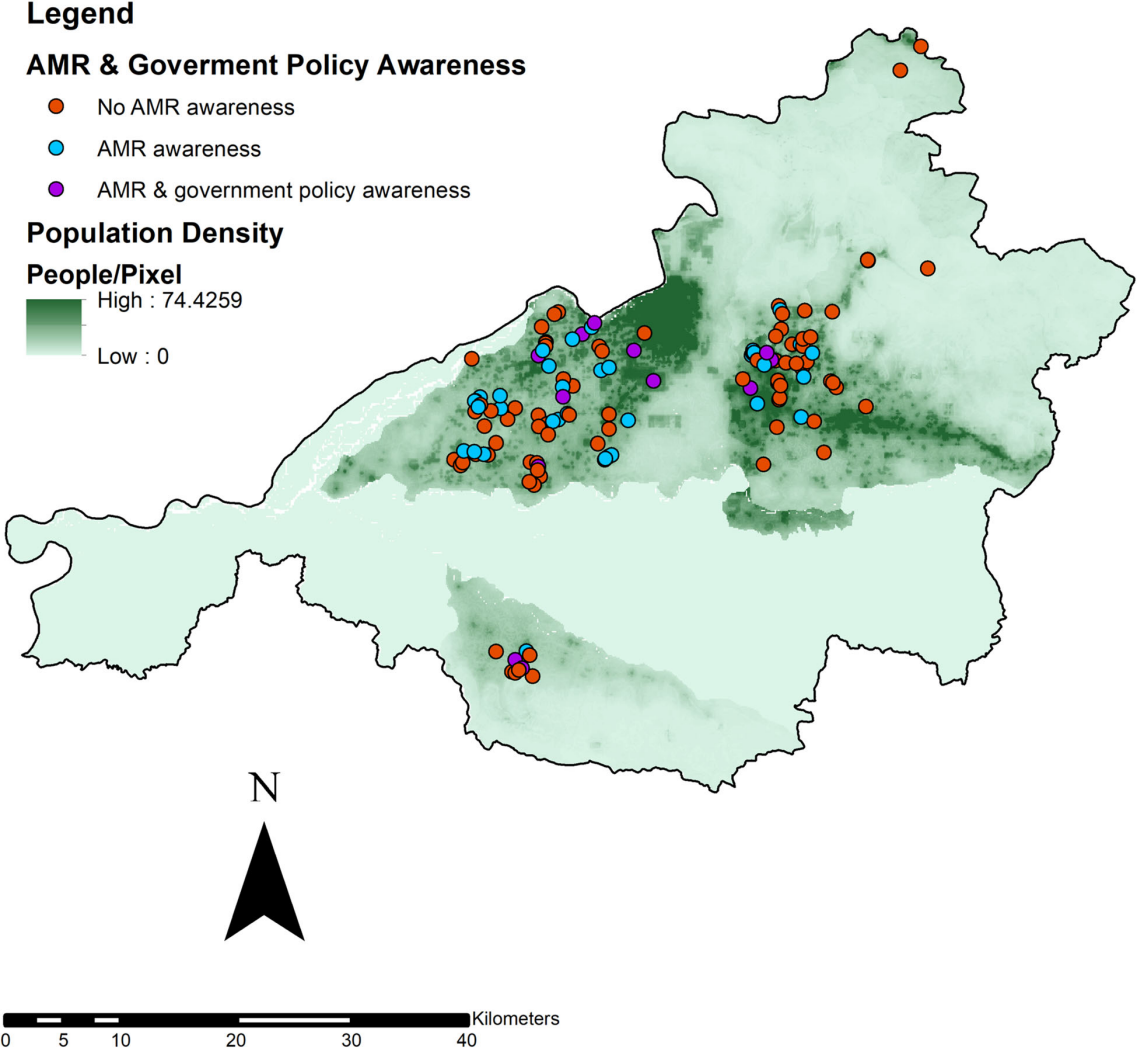
Map of Chitwan District, Nepal with participating commercial poultry farmers (*N* = 136), their AMR and government policy awareness levels, and the underlying human population density. 14 farms (9%) mapped outside the study area and were not included in this map, potentially due to mobile service issues in rural border areas of the district.1

Nepal’s centralized government has enacted specific policies to restrict the use of antimicrobials in livestock populations as a response to growing AMR concerns. Only 18% of producers could recall these policies, among whom were well-distributed across the Chitwan District (Fig. 1). Commercial poultry producers were asked a series of questions about antimicrobial use withdrawal periods, which are implemented to reduce human exposure and consumption of antimicrobial residues in animal meat. A majority (57%) of producers had previous knowledge of withdrawal periods, however, only 48% of these producers complied with withdrawal timelines.

### Antimicrobial use knowledge and practices

The majority of commercial poultry producers (99%) self-reported antimicrobial use throughout poultry production (Table 2). Most farmers (69%) reported use of antimicrobials in the past 3 months, while 22% reported use within the past week. Most producers (95%) cited disease treatment for antimicrobial use. Many also reported using antimicrobials for disease prevention (58%) and growth promotion (13%). Only one producer could not recall the reason for antimicrobial use during poultry production. In terms of antimicrobial administration routes, 94% of farmers dosed antimicrobials through drinking water, 30% enriched feed with antimicrobials, and 18% utilized injection routes. Sixty-eight percent of producers obtained antimicrobials for their poultry through veterinarian prescriptions. The remaining producers sometimes (29%), never (2%), and did not know (< 1%) if they received antimicrobials through veterinary drug prescriptions. Of those who treated their flocks with antimicrobials, most (65%) producers consulted a veterinarian when they suspected disease in their flocks, 24% consulted a veterinary technician, and 10% self-treated.

**Table 2:** Commercial poultry farmer antibiotic use knowledge and practices in Chitwan District Nepal, *N* = 150 farmers

Antibiotic Use Characteristic	Farmer No. (%)
**Use antibiotics to raise their poultry**	
Yes	148 (98.6)
No	1 (0.7)
Don’t know	1 (0.7)
**Used antibiotics in poultry production in past week**	
Yes	34 (22.7)
No	115 (76.6)
Don’t know	1 (0.7)
**Used antibiotics in poultry production in past 3 months**	
Yes	104 (69.3)
No	44 (29.3)
Don’t know	2 (1.4)
**Purposes of antibiotic use**	
Disease prevention	87 (58.0)
Growth promotion	20 (13.3)
Disease treatment	143 (95.3)
Don’t know	1 (0.7)
**Administration routes of antibiotics**	
Feed	46 (30.7)
Water	141 (94.0)
Injection	27 (18.0)
**Use of veterinary prescription for poultry antibiotics**	
Always	102 (68.0)
Sometimes	44 (29.3)
Never	3 (2.0)
Don’t know	1 (0.7)
**Knowledge of poultry withdrawal antibiotic period**	
Yes	85 (56.7)
No	65 (43.3)
**Knowledge and use withdrawal antibiotic period**^**a**^	
Yes	40 (47.6)
No	44 (52.4)
**Who do you consult when poultry are sick?**	
Veterinarian	97 (64.6)
Veterinary technician	36 (24.0)
Self-treat	15 (10.0)
Don’t treat	1 (0.7)
Don’t know	1 (0.7)

^a^ Missing data from one farmer

### Farmer antimicrobial knowledge and use in poultry production

Producers reported antimicrobial knowledge pertaining to brand names, antimicrobial classes, and non-antimicrobial supplements. Of the 149 producers who reported antimicrobial use during animal production, 148 could identify at least one antimicrobial. Producers reported using 26 unique drugs, which comprised 13 different classes and drug combinations (Table 3). Almost all producers (93%) self-reported usage of brands that utilize drug combinations, for example Colidox (Colistin + Doxycycline), Dimoxan (Colistin + Amoxicillin), Neodox (Neomycin + Doxycycline), and Sulphatrim (Sulfamethoxazole + Trimethoprim). Neodox, a combination of neomycin and doxycycline, was the highest reported drug combination used by 71% of poultry farmers. The antimicrobial drug class most frequently self-reported was polypeptides (35%), which was driven by the high use of the drug combination colistin (31%). The second most used antimicrobial family class was fluoroquinolones (30%), which includes ciprofloxacin, enrofloxacin, and levofloxacin.

**Table 3:** Antimicrobials used by farmers in commercial poultry production in Chitwan District Nepal, *n* = 150 farmers

Antibiotic Drug Class	Farmer No. ***N*** = 150 (%)	Self-Reported Antibiotic Drug	ATCvet Code	Farmer No. N = 150 (%)
Amphenicol	1 (0.7)	Chloramphenicol	QG01AA05	1 (0.7)
Aminoglycoside	35 (23.3)	Amikacin	QJ01GB06	8 (5.3)
		Gentamicin	QJ01GB03	26 (17.3)
		Neomycin	QJ01GB05	1 (0.7)
Beta-lactam	3 (2.0)	Amoxicillin	QJ01CA04	3 (2.0)
Cephalosporin	14 (9.3)	Cefalexin	QJ01DB01	11 (7.3)
		Cefuroxime	QJ01DC02	3 (2.0)
Fluoroquinolone	45 (30.0)	Ciprofloxacin	QJ01MA02	14 (9.3)
		Enrofloxacin	QJ01MA90	12 (8.0)
		Fluoroquinolone	QJ01MA	3 (2.0)
		Levofloxacin	QJ01MA12	13 (8.7)
Lincosamide	1 (0.7)	Lincomycin	QJ01FF02	1 (0.7)
Macrolide	32 (21.3)	Azithromycin	QJ01FA10	4 (2.7)
		Erythromycin	QJ01FA01	4 (2.7)
		Tylosin	QJ01FA90	24 (16.0)
Oxazolidinone	3 (2.0)	Furazolidone (Furasil)	QJ01XE90	3 (2.0)
Polypeptide	52 (34.7)	Bacitracin Methylene Disalicylate (BMD)	QJ01XX10	6 (4.0)
		Colistin	QJ01XB01	46 (30.7)
Pleuromutilin	5 (3.3)	Tiamulin	QJ01XQ01	5 (3.3)
Sulfonamide	7 (4.7)	Sulfonamide	QJ01EQ	7 (4.7)
Tetracycline	18 (12.0)	Chlortetracycline	QJ01AA03	8 (5.3)
		Doxycycline	QJ01AA02	10 (6.7)
Combination drug class formulations	139 (92.7)	Colidox (Colistin + Doxycycline)	QA07AA98	10 (6.7)
		Dimoxan (Colistin + Amoxicillin)	QA07AA98	12 (8.0)
		Neodox (Neomycin + Doxycycline)	QJ51RG01	107 (71.3)
		Sulphatrim (Sulfamethoxazole + Trimethoprim)	QJ01EW	10 (6.7)
Don’t know	1 (0.7)	–	–	1 (0.7)
Don’t use antibiotics	1 (0.7)	–	–	1 (0.7)

## Discussion

Antimicrobial use in Nepal’s animal agricultural sector may contribute to downstream AMR infections in humans and other animals through various transmission mechanisms, including direct contact between infected animals and humans, wastewater effluent, and consumption and handling of contaminated animal products. A One Health approach is needed to tackle AMR due to the interconnectedness of the use of antimicrobials in animal agriculture and human health. The Nepal government has enacted policies and antimicrobial campaigns to address the AMR challenge. However, little research has assessed the extent to which Nepal’s AMR initiatives have improved overall antimicrobial practices or monitored overall AMR trends in animal agriculture [14]. To the authors’ knowledge, this is the first study that has investigated production practices in Chitwan District’s commercial poultry industry to gain insight into antimicrobial-use and AMR knowledge.

This study demonstrated AMR knowledge gaps among Chitwan’s poultry producers. This lack of AMR knowledge and current policies that address responsible antimicrobial use demonstrates that despite efforts to improve stakeholder outreach from the Veterinary Standards and Drug Administration Office (VSDAO) and the Veterinary Public Health Office (VPHO), widespread policy awareness has yet to reach many producers [12, 15]. Effective dissemination is not a new challenge nor isolated to Nepal. Similar observations were reported in Vietnam among poultry producers who tended to score worse on AMR knowledge compared to individuals from other food animal sectors [16]. Gaps also existed regarding the use of critically important human antimicrobials in the poultry industry, such as colistin, fluoroquinolones, and chloramphenicol. The self-reported colistin use is especially concerning as it is a last-line antimicrobial for Gram-negative infections in human patients. Previous widespread use of colistin in animal agriculture propagated resistant bacterial infections in humans and animals, leading to its ban in many countries, including India and China [17–19]. The lack of AMR knowledge and judicious use, particularly regarding drugs of high consequence, may further perpetuate its development in food animals and humans in Nepal.

Over 10% of study participants indicated that they administered antimicrobials for growth promotion, a practice that has been adopted to theoretically increase animal mass for quicker turnout and higher net profits. This corresponds with subtherapeutic dosing, a major AMR contributor, and in Nepal may be responsible for up to 50% of contaminated retail poultry isolates that express multidrug resistant profiles [20, 21]. This study was not the first to have unearthed the problem of growth promotion use—other reports have illustrated similar injudicious uses of antimicrobials in the livestock sector within LMIC, including Nepal [14, 15, 22]. However, to the authors’ knowledge, no previous studies have evaluated AMR knowledge and husbandry practices of Nepalese poultry producers who use antimicrobials for growth promotion. Although only a relatively small percentage of respondents used antimicrobials for growth promotion (13%), human and animal health organizations have denounced all growth promotion use [23–25]. Nepal’s government has acknowledged the need for change, releasing the 2014 National Treatment Guidelines which supported a zero-tolerance policy for growth promotion uses of antimicrobials. In 2017 Nepal took further action to ban importation of antimicrobial enriched feed; however, this policy failed to eliminate in-country antimicrobial enrichment of animal feed. Nepal’s Ministry of Livestock Development has also promoted awareness of the issue through training and livelihood programs to educate youth interested in animal agriculture [26]. Unfortunately, limited resources have impacted the effectiveness of policy dissemination, enforcement, and monitoring [14, 26].

Abiding by withdrawal periods is critical to prevent consumer exposures to antimicrobial residues, and noncompliance with withdrawal periods may indicate lack of knowledge regarding residues, potential contribution to AMR, and general AMR experiences [27]. Only half of the producers surveyed in this study were familiar with withdrawal periods, and among those, only half complied with them. Prior research has demonstrated that the unfamiliarity with antimicrobial residues and withdrawal periods is common among Nepalese producers [15]. Disregard for withdrawal periods may result in antimicrobial residue contamination in the food supply that can expose consumers to unknown levels of antimicrobials. Several studies in Nepal have demonstrated antimicrobial residues in sampled meat ranging from 13% up to 62% [28, 29]. Although cooking temperatures destroy antimicrobial residues in meat, inadequately cooked meat may result in antimicrobial residue consumption and two major negative outcomes: antimicrobial resistance development and adverse drug reactions [21]. As mentioned previously, many producers administered colistin, and subtherapeutic ingestion of its residues may lead to resistance development and subsequent infections among individuals with direct poultry contact or through consumption of contaminated meat. In addition to colistin, other drug residues have been found in Nepal’s food supply, which is a cause for concern and a priority that Nepal’s federal agencies must address [15, 29]. Currently, the Ministry of Livestock Development and the Department of Livestock services share responsibility to respond to the withdrawal period awareness and observance deficiencies through the National Antimicrobial Surveillance Center [12].

Although Nepal has initiated steps to address AMR challenges through a policy infrastructure and educational awareness, these efforts do not appear to be adequately disseminated nor enforced to promote overall systemic change. Although policy and education campaigns are crucial, they alone may not solve all production practice issues [15, 30, 31]. Policies should consider banning targeted antimicrobials critical to human health, e.g., colistin, coupled with robust monitoring systems that measure antimicrobial uses, including grams of active ingredients and indications. These mechanisms have already been effective in reducing resistance in both humans and animals in countries like China [18, 32]. Nepal may also fill critical policy gaps such as those surrounding feed enrichment and the use of antimicrobials of high consequence for AMR and adverse reactions in humans. Policy accountability can be strengthened through targeted surveillance, consisting of a multifaceted approach to detect AMR infections, product contamination trends, antimicrobial use, and antimicrobial levels in feed. Improved outreach capacities, paired with increased veterinary resources and extensive monitoring in operations and in retail meat products, can create a framework for a regulatory enforcement structure.

### Strengths and limitations

This study provides unique access to commercial poultry operations in the highest product density district in Nepal to assess AMR and antimicrobial use knowledge and practices. This is the first study in Nepal to collect antimicrobial use data in commercial poultry through farmer reporting and farm visit confirmation of products used. This research fills a critical literature gap by assessing AMR knowledge in this frontline agricultural population. Study limitations include the self-reported data dependence and the cross-sectional study design. Unfortunately, self-reported antimicrobials could not all be confirmed through identification of packaging. Additionally, antimicrobial use may be a dynamic or seasonal practice on these farms, which were unable to be evaluated given the study design. Unfortunately, the frequency and dosage of the self-reported antimicrobials could not be ascertained, which is important since reported antimicrobials of high consequence may ultimately be used at a low frequency. Further research is needed to draw causal inferences between antimicrobial use on commercial poultry farms and local AMR epidemiology. It is important to fill AMR surveillance gaps in both food animal and human populations in Nepal and to work with the growing poultry production industry on antimicrobial use education and practice regulations. Due to landslide concerns in the Madi Municipality during the study duration, data from all commercial farms in Madi were retrieved over the phone. Another limitation was the inability to conduct interviews in remote locations; however, these phone interviews did not significantly bias the validity of the data collected from this specific district as all information could easily be ascertained remotely.

## Conclusions

AMR knowledge among Nepal’s poultry producers have resulted in poor antimicrobial use practices, including low antimicrobial withdrawal period compliance, significant use of antimicrobials for growth promotion, and the sustained use of critically important antimicrobials.. Outreach efforts in combination with increased veterinary resource allocations can educate and provide support for producers as to the benefits of antimicrobial stewardship. Expansive surveillance efforts to monitor antimicrobial use and residues in retail meat products may also create a framework for a regulatory enforcement structure to maintain accountability. Upfront investment in these activities is recommended to mitigate AMR and to promote the health of humans, animals, and the environment.

## Abbreviations

AMR: antimicrobial resistance
LMIC: lower- and middle-income country
MoLD: Ministry of Livestock Development
NAP-AMR: National Action Plan for Antimicrobial Resistance
VSDAO: Veterinary Standards and Drug Administration Office
